# A tissue specific-infection mouse model of SARS-CoV-2

**DOI:** 10.1038/s41421-023-00536-0

**Published:** 2023-04-20

**Authors:** Bo Yang, Chao Liu, Xiaohui Ju, Bingbing Wu, Zhuangfei Wang, Fucheng Dong, Yanying Yu, Xiaohui Hou, Min Fang, Fei Gao, Xuejiang Guo, Yaoting Gui, Qiang Ding, Wei Li

**Affiliations:** 1 Guangdong and Shenzhen Key Laboratory of Male Reproductive Medicine and Genetics, Institute of Urology, Peking University Shenzhen Hospital, Shenzhen PKU-HKUST Medical Center, Shenzhen, Guangdong China; 2 Guangzhou Women and Children’s Medical Center, Guangzhou Medical University, Guangzhou, Guangdong China; 3 State Key Laboratory of Stem Cell and Reproductive Biology, Institute of Zoology, Chinese Academy of Sciences, Beijing, China; 4 Center for Infectious Disease Research, School of Medicine, Tsinghua University, Beijing, China; 5 University of Chinese Academy of Sciences, Beijing, China; 6 Zunyi Medical University, Zunyi, Guizhou China; 7 CAS Key Laboratory of Pathogenic Microbiology and Immunology, Institute of Microbiology, Chinese Academy of Sciences, Beijing, China; 8 State Key Laboratory of Reproductive Medicine, Nanjing Medical University, Nanjing, Jiangsu China

**Keywords:** Mechanisms of disease, Biological techniques

## Abstract

Animal models play crucial roles in the rapid development of vaccines/drugs for the prevention and therapy of COVID-19, but current models have some deficits when studying the pathogenesis of SARS-CoV-2 on some special tissues or organs. Here, we generated a human ACE2 and SARS-CoV-2 N^F/F^ knockin mouse line that constitutively expresses human ACE2 and specifically expresses SARS-CoV-2 N gene induced by Cre-recombinase. By crossing with Cre transgenic lines allowing for lung-specific and constitutive expression, we generated lung-specific (Sftpc-hACE2-N^F/F^) and constitutive SARS-CoV-2 N (EIIa-hACE2-N^F/F^) expressing mice. Upon intranasal infection with a SARS-CoV-2 GFP/ΔN strain which can only replicate in SARS-CoV-2 N expressed cells, we demonstrated that both the Sftpc-hACE2-N^F/F^ and EIIa-hACE2-N^F/F^ mice support viral replication. Consistent with our design, viral replication was limited to the lung tissues in Sftpc-hACE2-N^F/F^ mice, while the EIIa-hACE2-N^F/F^ mice developed infections in multiple tissues. Furthermore, our model supports different SARS-CoV-2 variants infection, and it can be successfully used to evaluate the effects of therapeutic monoclonal antibodies (Ab1F11) and antiviral drugs (Molnupiravir). Finally, to test the effect of SARS-CoV-2 infection on male reproduction, we generated Sertoli cell-specific SARS-CoV-2 N expressed mice by crossing with AMH-Cre transgenic line. We found that SARS-CoV-2 GFP/ΔN strain could infect Sertoli cells, led to spermatogenic defects due to the destruction of blood-testis barrier. Overall, combining with different tissue-specific Cre transgenic lines, the human ACE2 and SARS-CoV-2 N^F/F^ line enables us to evaluate antivirals in vivo and study the pathogenesis of SARS-CoV-2 on some special tissues or organs.

## Introduction

The SARS-CoV-2 is the aetiological agent of coronavirus disease 2019 (COVID-19)^1–5^. Up to date, January 2023, 663 million people have been infected, and more than 6 million people have died worldwide^6^. The long-term and large-scale epidemic of SARS-CoV-2 characterized by widespread community transmission, while causing large numbers of asymptomatic, mild and long COVID cases^7–9^, has brought great pressure on the global public health.

Since its outbreak in late 2019, many animal models have played crucial roles in aiding the rapid development of vaccines/drugs for prevention and therapy, as well as understanding the pathogenesis of SARS-CoV-2 infection and immune responses of hosts^10^. Multiple COVID-19 animal models have been developed to date, such as non-human primates^11,12^, genetically modified mice^13–15^, AAV- or Ad5-transduced mice^16,17^, as well as Syrian hamster^18^, ferret^19^, poultry, and domestic animal models^20^. Nevertheless, the deficiencies have emerged, when facing with new requirements about studying the pathogenesis of SARS-CoV-2 in different tissues^10^. In addition, all of these animal models are restricted to Animal Biosafety Level 3 (ABSL-3) laboratories, which strongly hinders the study of SARS-CoV-2 and development of countermeasures.

Cre-loxP system is widely used for mammalian gene editing^21^. This system has enabled researchers to investigate genes of interest in a tissue/cell (spatial control) and/or time (temporal control) specific manner^22^. Previously, we had developed a transcription and replication-competent viral-like particles of SARS-CoV-2 (SARS-CoV-2 trVLP) system, which could recapitulate the entire viral life cycle in vitro at BSL-2 laboratory^23–25^. In this study, we combined the advantages of Cre-loxP system and trVLP system to construct a tissue-specific infection mouse model. First, we generated a human ACE2 and SARS-CoV-2 N^F/F^ knockin mouse line that constitutively expresses human ACE2 and tissue-specifically expresses SARS-CoV-2 N induced by Cre recombinase. By the lung-specific and constitutively expressed SARS-CoV-2 N mice, we demonstrated that the Sftpc-hACE2-N^F/F^ is a lung-specific infection model. Next, our model supports different SARS-CoV-2 variants strains infection, and it can be used to evaluate the effects of therapeutic monoclonal antibodies and antiviral drugs. Finally, we focused on the symptoms of long COVID-19 in the male reproduction system. By crossing with AMH-Cre mice that specifically express Cre-recombinase in Sertoli cells^26^, we constructed the AMH-hACE2-N^F/F^ mice. Through direct intra-testicular challenge with SARS-CoV-2 GFP/ΔN in AMH-hACE2-N^F/F^ mice, we found that SARS-CoV-2 infected Sertoli cells and led to impaired spermatogenesis due to the destruction of blood-testis barrier (BTB) in testis. In summary, we generated a human ACE2 and SARS-CoV-2 N^F/F^ knockin mouse line, which enables us to study the pathogenesis of SARS-CoV-2 on different special tissues or organs. And we further demonstrated that the infection of SARS-CoV-2 indeed results in testicular damage by disrupting the BTB in Sertoli cells. Thus, our mouse model provides a novel in vivo platform to study the pathogenesis of SARS-CoV-2 in some special tissues or organs at BSL-2 laboratory.

## Results

### Generation of an hACE2 and SARS-CoV-2-N conditional knockin mouse line

**Fig. 1:**
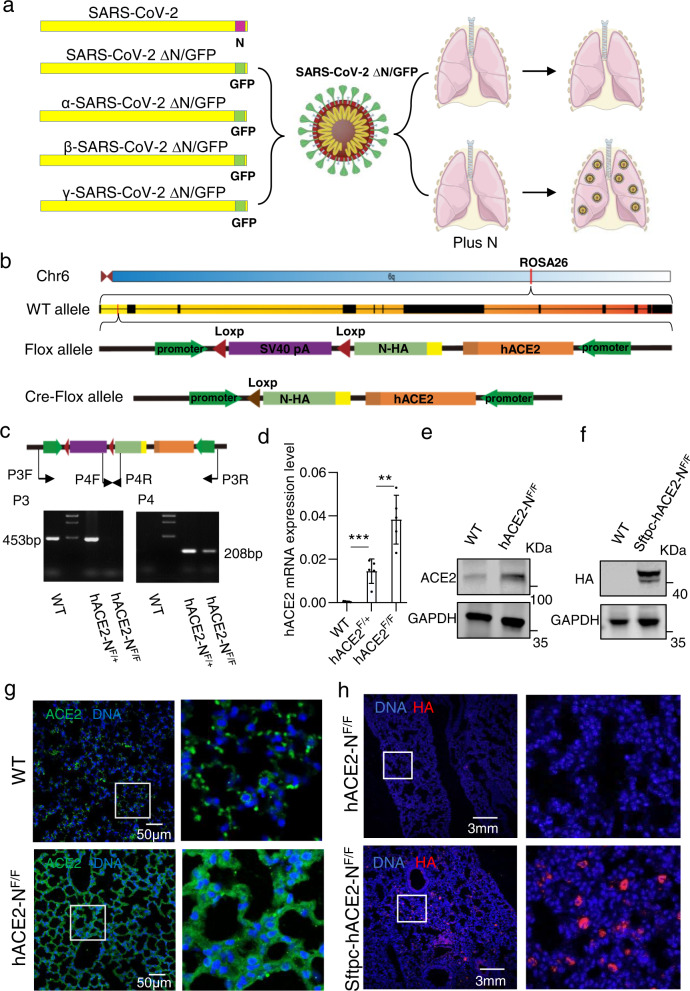
The design and construction of human ACE2 and SARS-CoV-2-N conditional knockin mouse. **a** The left sub-panel shows the genetic organizations of the SARS-CoV-2, SARS-CoV-2 GFP/ΔN, and related variants. The ORF encoding the N protein is replaced with GFP. The right sub-panel illustrates the concept that the SARS-CoV-2 GFP/ΔN virus can only replicate in lungs that are expressing the N protein. **b** The genetic sequences encoding the human ACE2 and SARS-CoV-2 N genes were inserted together into intron 1 of the *ROSA26* site located on chromosome 6. The PGK-Neo-6×SV40 pA sequence flanked by two loxP sites. When there were both the Cre recombinase and loxP sequences, the sequence between two loxP sites (PGK-Neo-6×SV40 pA) will be removed. **c** Primers used for genotyping are indicated in the cartoon. Two different primers are used to confirm the insertion element. **d** The expression of hACE2 in hACE2-N heterozygous, homozygous, and control mice (*n* = 5) assessed using qPCR. **e** Mouse lung tissue lysates were subjected to SDS PAGE followed by western blot with ACE2 antibody (recognize both human and mouse ACE2). **f** Mouse lung tissue lysates were subjected to SDS PAGE followed by western blot with HA antibody. **g** Immunofluorescence staining of lung sections with anti-ACE2 (green) antibody (recognize both human and mouse ACE2) and 4,6-diamidino-2-phenylindole (DAPI, blue) for evaluating hACE2 expressing cells in the lungs of hACE2-N^F/F^ mice. The two white frames are magnified at the right. **h** Immunofluorescence staining of lung sections with anti-HA (red) antibody and DAPI (blue) for evaluating SARS-CoV-2 N expressing cells in the lungs of Sftpc-hACE2-N^F/F^ mice. Two white frames are magnified at the right.

To study the pathogenesis of SARS-CoV-2 in different tissues, we generated a tissue-specific infection mouse model of SARS-CoV-2. We designed a conditional knockin mouse line capable of Cre recombinase-induced expression of both the human ACE2 protein and the SARS-CoV-2 N gene, which combined the advantages of Cre-loxP system and SARS-CoV-2 trVLP^23^. In this design, SARS-CoV-2 GFP/ΔN trVLP and related mutant strains are only able to replicate in tissues with the expression of the SARS-CoV-2 N gene (Fig. 1a). To generate the human ACE2 and SARS-CoV-2-N conditional knockin mouse line, we constructed a CAG promoter-loxP-PGK-Neo-6×SV40 pA-loxP-Kozak-SARS-CoV-2-N-HA-rBG pA-anti (CAG promoter-Kozak-Human ACE2 CDS-BGH pA) cassette (Fig. 1b), in which the cDNA encoding the human ACE2 protein was inserted downstream of the CAG promoter. A BGH poly (A) sequence was added to enhance mRNA stability. By flanking the PGK-Neo-6×SV40 pA sequence with two loxP sites, the expression of SARS-CoV-2 N gene could be induced by mating with a tissue-specific expressed Cre-transgenic mouse. And the construct was inserted into intron 1 of the ROSA26 site in mice.

Mice bearing the intended insertion were termed as hACE2-N^F/F^, and confirmed by PCR-based genotyping (Fig. 1c), and the expression of the human *ACE2* gene was confirmed by immunofluorescence staining, western blot, and qPCR (Fig. 1d, e, g). To confirm the efficiency of Cre-mediated SARS-CoV-2 N gene expression, we crossed hACE2-N^F/F^ mice with the Sftpc-Cre transgenic mice^27^ to obtain Sftpc-hACE2-N^F/F^ mice which expressed SARS-CoV-2 N in lung alveolar type II cells specifically. Sftpc-hACE2-N^F/F^ mice were initially administered tamoxifen (via intraperitoneal injection) daily for 7 days to induce Cre recombinase expression in the lung. Our results showed that the SARS-CoV-2 N-HA protein was expressed in lungs by immunofluorescence staining and western blot with an anti-HA antibody (Fig. 1f, h). Thus, we successfully generated a mouse line that constitutively expresses hACE2 and specifically expresses SARS-CoV-2 N in lung induced by Cre recombinase.

### Sftpc-hACE2-N^F/F^ mice are susceptible to intranasal infection of SARS-CoV-2 GFP/ΔN trVLP

**Fig. 2:**
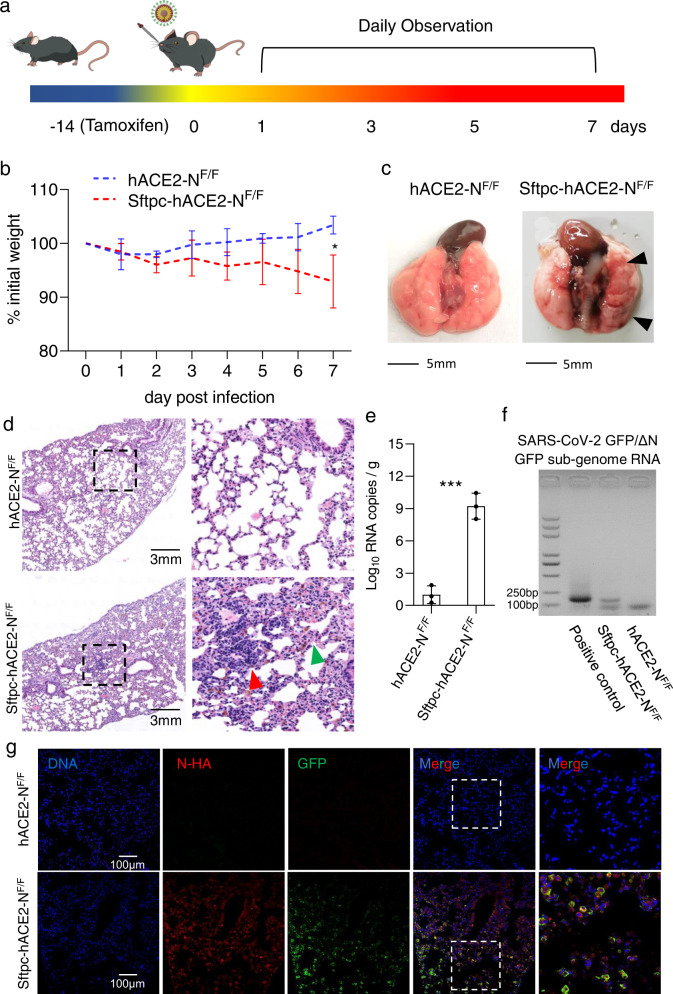
SARS-CoV-2 GFP/ΔN infection in Sftpc-hACE2-N^F/F^ mice. **a** A cartoon shows schedule of the intranasal infection. The Sftpc-hACE2-N^F/F^ mice were each intranasally infected with 1 × 10^5^ TCID_50_ of SARS-CoV-2 GFP/ΔN virus, and were sacrificed to collect tissue samples at 7 dpi. **b** Mouse body weights were monitored for up to 7 days (two experiments; *n* = 10; two-way ANOVA, symbols represent means ± s.e.m.). **c** Post-mortem examinations showed focal dark-red lesions throughout the dorsal region of the right middle lobe of the lung. **d** Pathological changes in Sftpc-hACE2-N^F/F^ mouse lung after infection. Five euthanized mice were used to examine the pathological changes in the lungs after 7 dpi. Mouse lung showed multifocal lesions with inflammatory infiltration (red arrow) and fibroplasia (green arrow). **e** Viral RNA was quantified using qPCR of mouse lung tissues collected at 7 dpi. **f** SARS-CoV-2 GFP/ΔN sub-genome monitored by using SARS-CoV-2 GFP RNA-specific primers. **g** Immunofluorescence analysis of mouse lung paraffin sections staining for SARS-CoV-2 GFP/ΔN GFP protein (green), N-HA (red), and DAPI (blue).

To test whether the mouse expressing SARS-CoV-2 N supports SARS-CoV-2 GFP/ΔN trVLP replication, tamoxifen was administered for 7 times in a total of 7 consecutive days via intraperitoneal injection before 14 days of the infection, after another 7-day recovery without tamoxifen treatment, 1 × 10^5^ 50% Tissue Culture Infectious Dose (TCID_50_) of SARS-CoV-2 GFP/ΔN trVLP was intranasally inoculated into Sftpc-hACE2-N^F/F^ mice. The control mice hACE2-N^F/F^ received the same dose of SARS-CoV-2 GFP/ΔN trVLP. Mouse body weights were then monitored daily for up to 7 days, and mice were sacrificed to collect tissue samples at 7 days post-infection (dpi) (Fig. 2a). The Sftpc-hACE2-N^F/F^ mice displayed significant weight loss at 7 dpi compared with control groups (Fig. 2b). Viral RNA replication was detected in lungs from trVLP infected Sftpc-hACE2-N^F/F^ mice, whereas no detectable viral RNA was observed in lungs from control mice (Fig. 2e).

As the N gene was replaced with green fluorescent protein (GFP) gene in the SARS-CoV-2 GFP/ΔN genome^23^, we also monitored GFP RNA using SARS-CoV-2 GFP/ΔN sub-genome specific primers (Fig. 2f) and detected GFP protein accumulation in lungs of mouse infected by SARS-CoV-2 GFP/ΔN with immunostaining (Fig. 2g). Compared with the hACE2-N^F/F^ mice, we found that the GFP RNA and protein were only detected in the Sftpc-hACE2-N^F/F^ mice lung samples. To test the safety of SARS-CoV-2 GFP/ΔN trVLP produced from infected Sftpc-hACE2-N^F/F^ mice, we performed the experiments of animal infection and genetic stability of SARS-CoV-2 GFP/ΔN trVLP. We found that the SARS-CoV-2 GFP/ΔN trVLP produced from infected Sftpc-hACE2-N^F/F^ mice could not infect hACE2 mice (Supplementary Fig. S1c), and the genetic stability experiments showed that no PCR product of >1050 bp was detected in any of the samples (Supplementary Fig. S1d), which is similar to the SARS-CoV-2 GFP/ΔN trVLP produced from cells^23^. These results demonstrated that the Sftpc-hACE2-N^F/F^ mice support SARS-CoV-2 GFP/ΔN trVLP replication in lung tissues in a relative safe way.

### Sftpc-hACE2-N^F/F^ mice developed interstitial pneumonia upon SARS-CoV-2 GFP/ΔN infection

Having found that Sftpc-hACE2-N^F/F^ mice can support SARS-CoV-2 GFP/ΔN trVLP replication in lung tissues, we subsequently examined whether Sftpc-hACE2-N^F/F^ mice develop interstitial pneumonia upon SARS-CoV-2 GFP/ΔN infection. To check whether the SARS-CoV-2 GFP/ΔN infected Sftpc-hACE2-N^F/F^ mice produced similar pathological features as other genetically modified mice^14^, histopathological examination was performed in lung sections from those animals. In the SARS-CoV-2 GFP/ΔN infected Sftpc-hACE2-N^F/F^ mice, post-mortem examinations showed focal dark-red lesions throughout the dorsal region of the right middle lobe of the lung (Fig. 2c), and hematoxylin-eosin (H&E) staining showed that the Sftpc-hACE2-N^F/F^ mice developed interstitial pneumonia characterized by inflammatory cell infiltration and alveolar septal thickening when compared with wild type (WT) mice, uninfected Sftpc-hACE2-N^F/F^ mice, and SARS-CoV-2 GFP/ΔN infected hACE2-N^F/F^ mice (Fig. 2d; Supplementary Fig. S1a–b). Inflammatory cell infiltration was also detected in an immunohistochemistry (IHC) analysis (Supplementary Fig. S2a). In addition, we conducted qPCR-based cytokine profiling and found that compared with lungs from control mice, the lungs of SARS-CoV-2 GFP/ΔN infected Sftpc-hACE2-N^F/F^ mice had elevated cytokine levels (e.g., *Cxcl10*, *Il1b*, *Ifng*, *ccl2*, *Ifnb1*, *Cxcl11*, and *Il6*, among others) (Supplementary Fig. S2b). These data support that upon SARS-CoV-2 GFP/ΔN infection, Sftpc-hACE2-N^F/F^ mice develop interstitial pneumonia.

### Sftpc-hACE2-N^F/F^ mice support SARS-CoV-2 variants infection

**Fig. 3:**
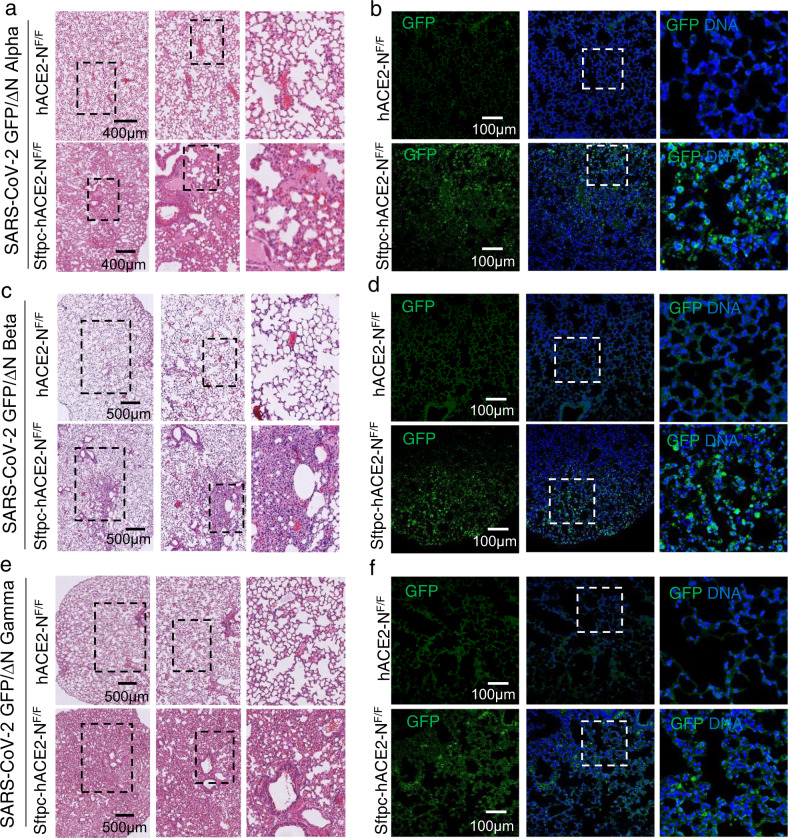
SARS-CoV-2 GFP/ΔN variants can infect Sftpc-hACE2-N^F/F^ mice. **a**, **c**, **e** H&E staining shows the pathological changes in Sftpc-hACE2-N^F/F^ mouse lung after infection with SARS-CoV-2 GFP/ΔN Alpha (**a**), Beta (**c**), Gamma (**e**) variants. The dark frame blocks were magnified on the right. **b**, **d**, **f** Immunofluorescence staining of mouse lungs for SARS-CoV-2 GFP/ΔN GFP protein (green), and DAPI (blue).

Due to the emergence of several variants throughout the world^28,29^, we further constructed SARS-CoV-2 GFP/ΔN related variants. To assess if the SARS-CoV-2 GFP/ΔN variants can replicate in Sftpc-hACE2-N^F/F^ mice, we infected Sftpc-hACE2-N^F/F^ mice with SARS-CoV-2 GFP/ΔN variants and found that all the SARS-CoV-2 GFP/ΔN variants could replicate in Sftpc-hACE2-N^F/F^ mice. Immunostaining of lung sections from the infected mice showed that viral GFP protein could be detected in samples from infected Sftpc-hACE2-N^F/F^ mice (Fig. 3b, d, f). Results from H&E staining lung sections from Sftpc-hACE2-N^F/F^ mice infected with different SARS-CoV-2 GFP/ΔN variants showed that they developed interstitial pneumonia in lung tissues (Fig. 3a, c, e). Therefore, we can study the pathogenesis of SARS-CoV-2 variants using this mouse model.

### Sftpc-hACE2-N^F/F^ infection model is a lung-specific infection model

**Fig. 4:**
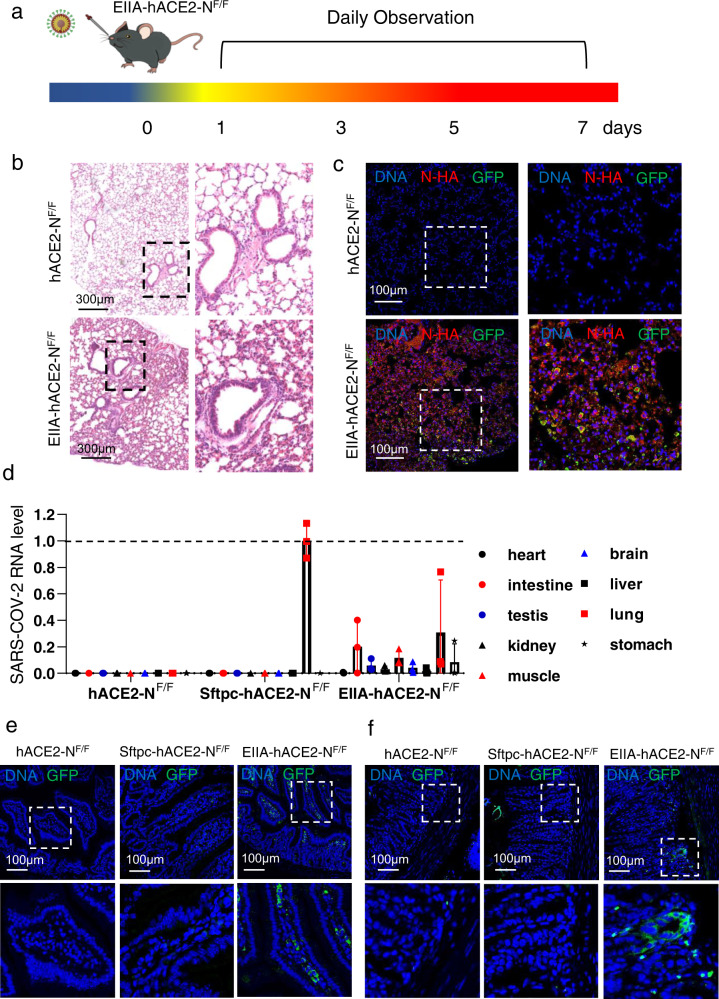
SARS-CoV-2 GFP/ΔN infection in EIIA-hACE2-N^F/F^ mice. **a** EIIA-hACE2-N^F/F^ mice were intranasally challenged with 1 × 10^5^ TCID_50_ virus each and sacrificed to collect tissue samples at 7 dpi. **b** Pathological changes in lungs of EIIA-hACE2-NF/F mice after infection. Five euthanized mice were used to examine the pathological changes in the lungs at 7 dpi. **c** Immunofluorescence staining of mouse lung paraffin sections against the SARS-CoV-2 GFP/ΔN GFP protein (green), N-HA (red), and DAPI (blue). **d** Tissue distribution of SARS-CoV-2 viral RNA (the *GFP* gene RNA) level which relative to the Sftpc-hACE2-N mouse lung tissue group. Each tissue was processed with viral RNA copies analysis by real-time qPCR. **e**, **f** Immunofluorescence staining of mouse intestine (**e**) and stomach (**f**) paraffin sections for SARS-CoV-2 GFP/ΔN GFP protein (green) and DAPI (blue).

After confirming that Sftpc-hACE2-N^F/F^ mice are susceptible to SARS-CoV-2 GFP/ΔN infection and developed interstitial pneumonia, we focused on the question whether the Sftpc-hACE2-N^F/F^ infection model is a lung-specific infection model. To answer this question, we constructed a constitutively expressed SARS-CoV-2 N mice by crossing with EIIa-Cre line^30^, which we termed as EIIa-hACE2-N^F/F^, and it expressed in a wide range of tissues, including the lung (Supplementary Fig. S3a). Compared with EIIa-hACE2-N^F/F^ infected mice, we can test and verify whether the Sftpc-hACE2-N^F/F^ is a lung-specific infection model. The EIIa-hACE2-N^F/F^ mice were intranasally challenged with 1 × 10^5^ TCID_50_ virus each, and sacrificed to collect tissue samples at 7 dpi (Fig. 4a).

We initially tested whether EIIa-hACE2-N^F/F^ mice are also susceptible to infection of SARS-CoV-2 GFP/ΔN trVLP. In comparison with WT mice, uninfected EIIa-hACE2-N^F/F^ mice, and SARS-CoV-2GFP/ΔN infected hACE2-N^F/F^ mice, H&E staining showed that only infected EIIa-hACE2-N^F/F^ mice developed interstitial pneumonia (Fig. 4b; Supplementary Fig. S4). As expected, immunostaining of lung sections from the infected EIIa-hACE2-N^F/F^ mice showed GFP protein expression in lung cells (Fig. 4c). Then, we tested whether the Sftpc-hACE2-N^F/F^ mice get lung-specific infection through compared with infected EIIa-hACE2-N^F/F^ mice. Based on the results, SARS-CoV-2 GFP/ΔN GFP RNA was found in the lung tissues from both Sftpc-hACE2-N^F/F^ and EIIa-hACE2-N^F/F^ mice, whereas the viral GFP RNA was observed in the intestine, stomach, and testis from EIIa-hACE2-N^F/F^ mice (Fig. 4d). In addition, immunostaining of intestine and stomach sections from the infected mice showed that viral GFP protein expression could only be detected in samples from EIIa-hACE2-N^F/F^ mice (Fig. 4e, f). A mosaic pattern of SARS-CoV-2 N expression has been commonly observed in EIIa-hACE2-N^F/F^ mouse lung (Supplementary Fig. S3a), and we speculated that the lower viral RNA level in EIIa-hACE2-N^F/F^ lung compared with that of Sftpc-hACE2-N^F/F^ mice may be caused by the SARS-CoV-2 N-HA expression level. To test this possibility, we detected the expression level of SARS-CoV-2 N-HA in EIIa-hACE2-N^F/F^ and Sftpc-hACE2-N^F/F^ lung tissues. The western blot results revealed that the SARS-CoV-2 N-HA protein level in EIIa-hACE2-N^F/F^ mice lung tissues is indeed lower than that in Sftpc-hACE2-N^F/F^ mice lung tissues (Supplementary Fig. S3b). These results documented that Sftpc-hACE2-N^F/F^ is a lung-specific infection model, and this model can be used to generate different tissue-specific infection model through crossing with various Cre transgenic lines.

### Application of this model in evaluating the efficacy of antibodies and drugs

**Fig. 5:**
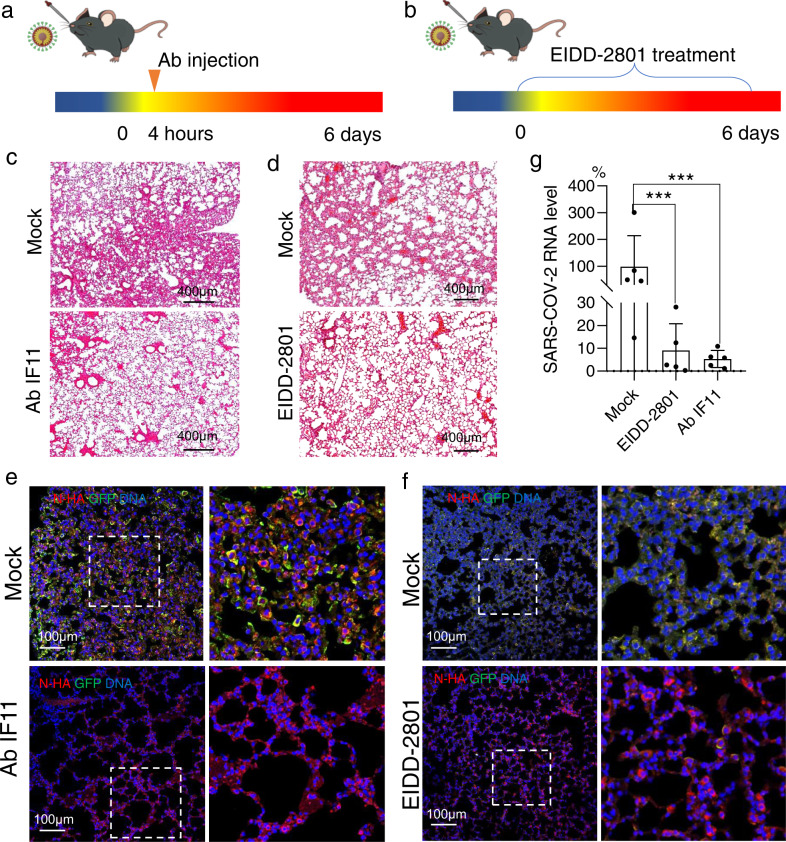
Therapeutic antibodies and antiviral drugs restrict viral infection in Sftpc-hACE2-N^F/F^ mice. **a**, **b** Sftpc-hACE2-N^F/F^ mice were intranasally infected with 1 × 10^5^ TCID_50_ virus and euthanized to collect tissue samples at 6 dpi. **a** Equivalent numbers of 10-week-old male and female sftpc-hACE2-N^F/F^ mice were administered vehicle or antibodies (Ab1F11) beginning at +4 h by intraperitoneal injection (*n* = 10 per group). Mice were intranasally infected with 1 × 10^5^ TCID_50_ SARS-CoV-2 GFP/ΔN strain. **b** Animals were orally administered 1 μg/kg EIDD2801 or vehicle before infection. Animals were euthanized at 6 dpi and lungs collected. **c** H&E staining shows the lung tissues of PBS or Ab1F11-treated mice. **d** H&E staining shows the lung tissues of vehicle and 1μg/kg EIDD 2801-treated mice. **e** Immunofluorescence staining shows mouse lungs from vehicle and Ab1F11-treated mice. **f** Immunofluorescence staining shows mouse lungs from vehicle and 1 μg/kg EIDD 2801-treated mice. SARS-CoV-2 GFP/ΔN GFP protein (green), and DAPI (blue). **g** Viral RNA was quantified by qPCR of mouse lung tissues from EIDD 2801-treated or Ab1F11-treated mice.

To test whether our models could be used for evaluation of antivirals, we verify the antiviral effects of a neutralizing antibody 1F11^31^ and molnuporavir^32^, which are well characterized with their antiviral activity against SARS-CoV-2 infection. At 4 h after intranasal infection with 1 × 10^5^ TCID_50_ of SARS-CoV-2 GFP/ΔN trVLP, Sftpc-hACE2-N^F/F^ mice were administered with vehicle (PBS) or the antibody (Ab1F11) (Fig. 5a). For the prophylactic orally administered EIDD-2801 experiment, we also performed in Sftpc-hACE2-N^F/F^ mice where we administered vehicle (10% polyethylene glycol (PEG) and 2.5% Cremophor RH 40 in water) or EIDD-2801 2 h prior to intranasal infection with 1 × 10^5^ TCID_50_ of SARS-CoV-2 GFP/ΔN trVLP, then vehicle or drug every 12 h thereafter (Fig. 5b). After treatment with 1F11 or EIDD-2801, H&E staining of lung tissues showed that both 1F11 and EIDD-2801 reduced the extent of lung hemorrhaging and diffuse alveolar damage as compared to vehicle-treated animals (Fig. 5c, d). Immunofluorescence staining of lung samples and qPCR results showed that both 1F11 and EIDD-2801 treatments resulted in reduced viral loads (Fig. 5e–g). These results indicated that our mouse model can be used as an in vivo platform under BSL-2 conditions to evaluate the antiviral effect of monoclonal antibodies and antiviral drugs^23^.

### Testicular damage induced by SARS-CoV-2 infection

**Fig. 6:**
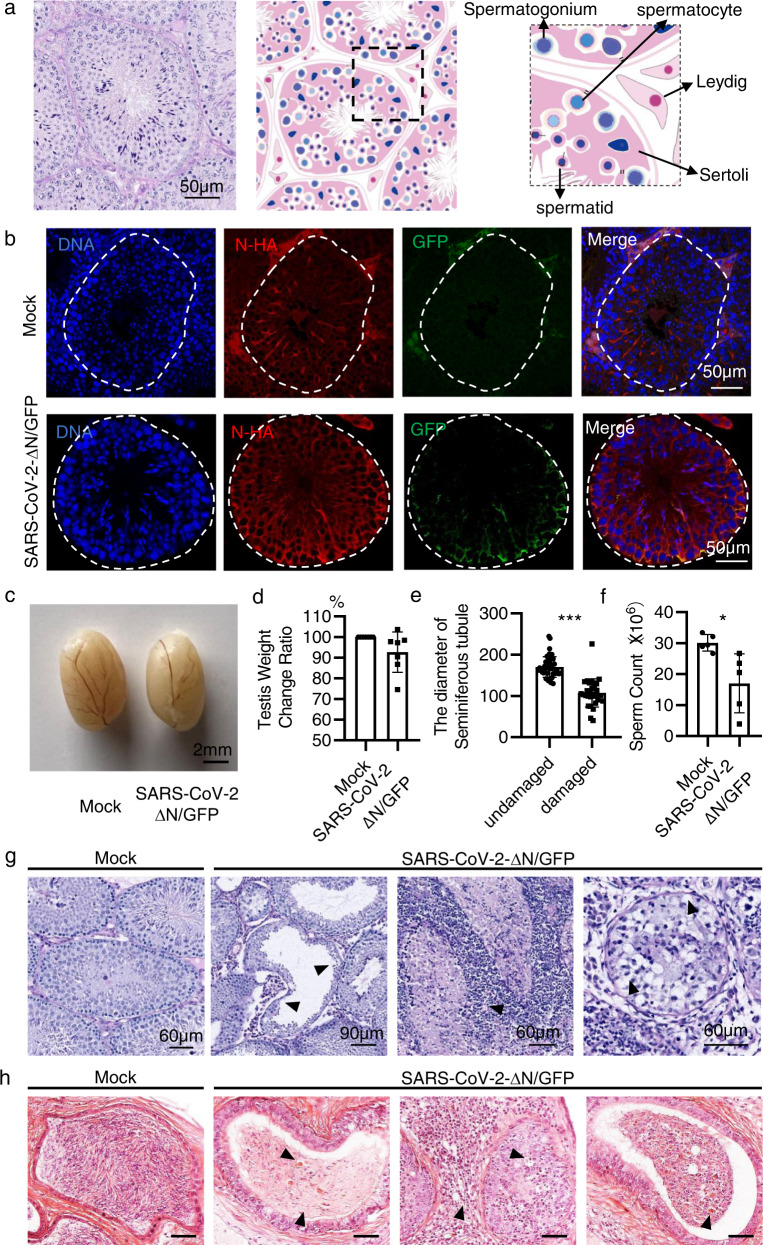
Spermatogenic defects of the AMH-hACE2-N^F/F^ mice after SARS-CoV-2 GFP/ΔN infection. **a** Illustration and representative H&E images of testicular section of WT mice show properly arranged testicular structure, seminiferous tubules and different cell types including Sertoli cells and germ cells at various stages. **b** Immunofluorescence staining of SARS-CoV-2 GFP in AMH-hACE2-N^F/F^ mouse testes. **c** Representative image of testis dissected from mock and SARS-CoV-2 GFP/ΔN-infected AMH-hACE2-N^F/F^ mouse. **d** Average weight of testis (*n* = 5–10 for each group). Error bars represent mean ± SD. **e** The diameter of seminiferous tubules. **f** Sperm counts. The epididymis was dissected, sperms were rinsed out for counting the number per testicle (*n* = 5–10 for each group). Error bars represent mean ± SD. *P < 0.05, **P < 0.01 by *t*-test comparing to the controls. **g** Histopathological changes of testis from SARS-CoV-2 GFP/ΔN -infected AMH-hACE2-N^F/F^ mice and control mice. **h** Representative H&E images of epididymis of mock and SARS-CoV-2 GFP/ΔN infected AMH-hACE2-N^F/F^ mice.

The aforementioned studies have demonstrated that the application of our mouse model in drug evaluation, we next sought to apply this model in studying the effect of SARS-CoV-2 infection on specific tissues or organs. As human *ACE2* is highly expressed in Sertoli cells and Leydig cells of testis^33–35^, it is possible that SARS-CoV-2 infects testis and leads to testicular damage. Although relevant studies have been reported^36–42^, the effect of SARS-CoV-2 infection on testis is still controversial^43,44^. The somatic Sertoli cells within the seminiferous tubules play a key role in supporting the maturation of germ cells (Fig. 6a)^45^. To investigate the effect of SARS-CoV-2 infection on testis, we generated AMH-hACE2-N^F/F^ mice that specifically expressed SARS-CoV-2 N in Sertoli cells^26^ (Supplementary Fig. S5).

**Fig. 7:**
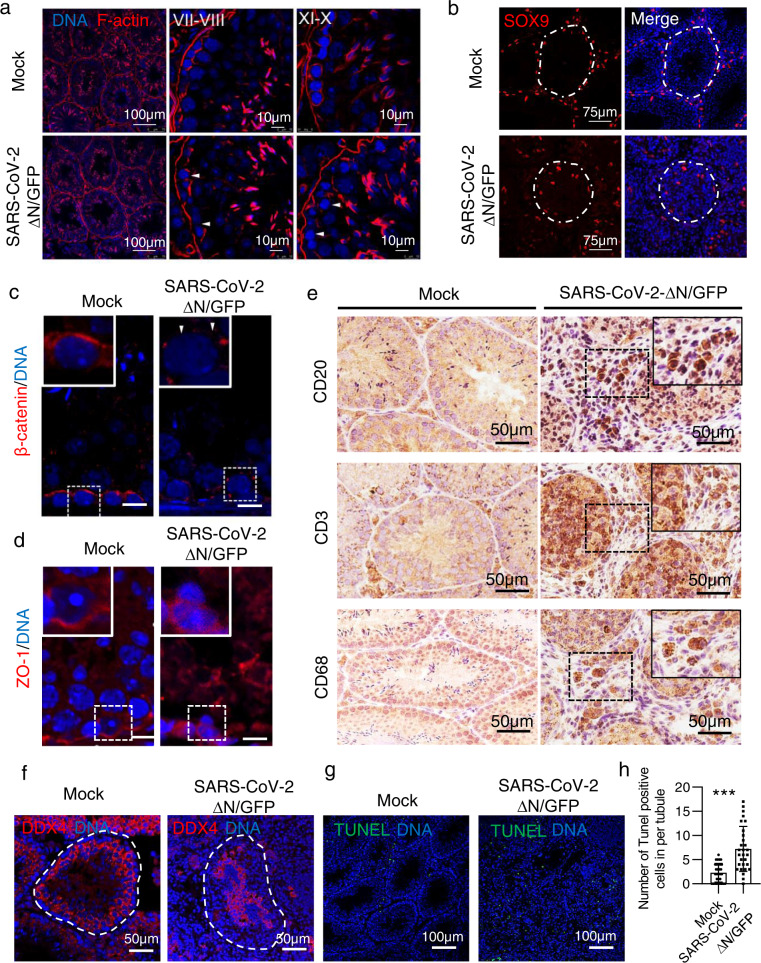
The integrity of BTB is disrupted in SARS-CoV-2 infected testes. **a** Distribution of F-actin in the testes of SARS-CoV-2 GFP/ΔN-infected AMH-hACE2-N^F/F^ mouse and control mouse, nuclei were stained with DAPI. **b** Immunofluorescence staining of SOX9 in adult mouse testes shows the abnormal distribution of Sertoli cells in SARS-CoV-2 GFP/ΔN-infected AMH-hACE2-N^F/F^ mouse and control mouse. **c**, **d** Immunofluorescence staining of β-catenin (**c**) and ZO-1 (**d**) in the adult mouse testes shows their abnormal distribution in SARS-CoV-2 GFP/ΔN-infected AMH-hACE2-N^F/F^ mouse and control mouse. **e** IHC staining analysis for B cells (CD20), T cells (CD3) and macrophages (CD68^+^) in SARS-CoV-2 GFP/ΔN infected AMH-hACE2-N^F/F^ mouse testes. **f** Immunofluorescence staining of DDX4 in the adult mouse testes shows abnormal distribution of germ cells in SARS-CoV-2 GFP/ΔN-infected AMH-hACE2-N^F/F^ mouse and control testes. **g** Representative images of apoptotic cells revealed by TUNEL in SARS-CoV-2 GFP/ΔN infected AMH-hACE2-N^F/F^ mouse testes and control groups. **h** The number of TUNEL-positive cells per seminiferous tubule.

Following intra-testicular inoculation with 5 × 10^3^ TCID_50_ SARS-CoV-2 GFP/ΔN trVLP in AMH-hACE2-N^F/F^ mice, immunofluorescence staining of viral GFP protein showed a few positive cells in seminiferous tubules at 4 dpi (Fig. 6b). At 7 dpi, there were no gross changes in the appearance of the testes or in the size of inoculated testes compared with the contralateral control testes (Fig. 6c, d). However, the sperm counts were significantly reduced in the SARS-CoV-2 GFP/ΔN infected testes (Fig. 6f, h), and severe testicular damage was observed (Figs. 6e, g, 7f–h; Supplementary Fig. S8b). Some seminiferous tubular lumens only contained cell debris (Fig. 6g; Supplementary Fig. S6a). Cytoplasmic vacuolation, degeneration, detachment of Sertoli cells into the lumen (Figs. 6g, 7b; Supplementary Fig. S6a–b), and mononuclear cell infiltration in testis (Fig. 6g; Supplementary Fig. S6a) were also observed. The epididymis had interstitial mononuclear cell infiltration with lumens filled with sloughed germ cells and cell debris (Fig. 6h). At 23 dpi, histological analysis of the SARS-CoV-2 GFP/ΔN infected AMH-hACE2-N^F/F^ testes showed severe damage of the seminiferous tubule structures with the loss of the central ductal lumen (Supplementary Fig. S7).

### SARS-CoV-2 infection disrupts the integrity of BTB

The BTB is formed between Sertoli cells, where it creates a unique microenvironment for spermatogenesis^46^. If BTB is disrupted, spermatogenesis can be severely impaired, and germ cells will loss due to cell death^47^, which is similar to the phenotype in SARS-CoV-2 GFP/ΔN infected AMH-hACE2-N^F/F^ testes. Then we tested the integrity of the BTB. According to the immunofluorescence results, F-actin was affected at VII-VIII and X-XI stages (Fig. 7a), suggesting that the BTB structure was disrupted in SARS-CoV-2 GFP/ΔN infected testes. SOX9 staining indicated that the Sertoli cells detached from the basal membrane (Fig. 7b). In the mock testes, tight junction protein ZO-1, adhesion junction proteins, and β-catenin were detected at the peripheral region of seminiferous tubules, where tight junctions are formed (Fig. 7c, d; Supplementary Fig. S8a). In contrast, the expressions of these proteins were significantly reduced in SARS-CoV-2 GFP/ΔN infected testes, and their distributions were also perturbed (Fig. 7c, d; Supplementary Fig. S8a). Moreover, IHC staining showed that there were increased immune cell infiltration (CD3 T cells, CD20 B cells, and CD68 macrophages) in the testicular interstitium compared with that of the control testis (Fig. 7e), and the TUNEL staining showed that the number of cell death in testis were dramatically increased (Fig. 7g, h). The immunofluorescence staining of DDX4 and γ-H2AX showed that spermatogenesis was severely impaired (Fig. 7f, Supplementary Fig. S8b). Together, our results revealed that Sertoli cells can be infected by SARS-CoV-2 GFP/ΔN, and the infection disrupts the integrity of the BTB, which eventually leads to oligospermia or even azoospermia.

## Discussion

To accelerate SARS-CoV-2 study, we generated a human ACE2 and SARS-CoV-2-N conditional knockin mouse line in which SARS-CoV-2-N expression is under the control of Cre-loxP system. By using the lung-specific and constitutively expressed Cre lines, we constructed Sftpc-hACE2-N^F/F^ and EIIa-hACE2-N^F/F^ mice. Further research demonstrated that both Sftpc-hACE2-N^F/F^ and EIIa-hACE2-N^F/F^ mice are susceptible to SARS-CoV-2 GFP/ΔN and the former is a lung-specific infection model. Moreover, we demonstrated that our model can be used to study the pathogenesis of different SARS-CoV-2 variants, and it can serve as an in vivo model to evaluate the effect of neutralizing antibodies and antiviral drugs under ABSL-2 condition.

The initial site of SARS-CoV-2 infection and replication is the sinonasal airway epithelium^48^. As the disease spreads down to the alveolar compartment, the primary cell being infected by SARS-COV-2 is the alveolar type II (AT2) cell, which is also the main cell type that expresses ACE2 and TMPRSS2 in the lung^49^. After SARS-CoV-2 infection, AT2 cells release the virus that infects AT2 cells, and also secrete interferons and inflammatory cytokines and chemokines to initiate the innate immune response. The inflammatory response includes mobilization of immune cells and tissue damage. The ultimate consequence is diffuse alveolar injury with loss of functional surfactant, damage of alveolar type I cells and endothelial cells, alveolar flooding and influx of inflammatory cells^50^. In Sftpc-hACE2-N^F/F^ mice, SARS-CoV-2 mainly replicates in AT2 cells, which can roughly mimic the lung pathogenesis infected by real virus (Fig. 2), suggesting that AT2 cells might be the major target of SARS-CoV-2 in lung tissues.

Although several hACE2 transgenic mice have been generated with different strategies^14^, our mouse model has several advantages. First, our model is safer than the previous mouse models, because the SARS-CoV-2 GFP/ΔN trVLP can only replicate in the N expressed mouse, it can be operated in the ABSL-2 condition, which could accelerate our studies on SARS-CoV-2. Second, since this model can be used to evaluate neutralizing antibodies and antiviral drugs, it will be a useful platform to test new vaccines and other potential therapeutics under ABSL-2 condition. Third, with the help of some Cre recombinase transgenic lines, the N gene can be induced to express in certain special cells, tissues or organs, such as lung, kidney, liver, spleen, and testis. These tissue-specific mouse models can be used to test whether SARS-CoV-2 infection directly or indirectly causes the post-COVID conditions in different tissues, and further dissect the underlying mechanisms. For example, we generated a Sertoli cell-specific mouse model to test the effect of SARS-CoV-2 infection on spermatogenesis by crossing with AHM-Cre mouse (Fig. 6b). We found that the SARS-CoV-2 GFP/ΔN indeed can infect the Sertoli cells. At 7 dpi, the germ cells lost due to cell death in SARS-CoV-2 GFP/ΔN infected AMH-hACE2-N^F/F^ testes (Fig. 6g). In addition, we provided evidence that the infection of SARS-CoV-2 disrupts the BTB (Fig. 7). Those results demonstrated that SARS-CoV-2 infection is a risk factor threatening BTB integrity, and causes testicular damage.

It is well known that a broad range of virus families, including human immunodeficiency virus, mumps virus, influenza, Zika virus, Coxsackie virus, may induce orchitis and even result in male infertility^51^. Our study demonstrates that SARS-CoV-2 infection influences spermatogenesis and provides direct evidence that SARS-CoV-2 infection may disrupt BTB integrity. However, we do not know how SARS-CoV-2 infection leads to BTB disruption. For this question, one hypothesis is that SARS-CoV-2 infection leads to Sertoli cells death or the BTB-related genes changes. Another hypothesis is that the immune effects of SARS-CoV-2 infection leads to testis damage such as the disruption of BTB integrity. The detailed molecular mechanisms of SARS-CoV-2 infection-causing to testis damage still need further study.

## Materials and methods

### Generation of human ACE2 and SARS-CoV-2-N ROSA26 conditional knockin mice

The gRNA to mouse *ROSA26* gene, the donor vector containing “CAG promoter-loxP-PGK-Neo-6×SV40 pA-loxP-Kozak-SARS-CoV-2-N-HA-rBG pAanti (EF1A promoter-Kozak-Human ACE2 CDS-BGH pA)” cassette, and Cas9 mRNA were co-injected into fertilized mouse eggs to generate targeted conditional knockin offspring. F0 founder animals were identified by PCR followed by sequence analysis, which were bred to wild-type mice to test germline transmission. All of the animal experiment were performed according to approved institutional animal care and use committee (IACUC) ptotocols (#2021-002) of the Institute of Zoology, Chineses Academy of Sciences.

### Cell culture

Caco-2 N and Caco-2 cells were maintained in Dulbecco’s modified Eagle medium (DMEM) (Gibco, China) supplemented with 10% (vol/vol) fetal bovine serum (FBS), and 50 IU/mL penicillin streptomycin in a humidified 5% (vol/vol) CO_2_ incubator at 37 °C. All cell lines were tested negative for mycoplasma.

### RNA isolation and RT-qPCR

Total cellular RNA was isolated using TRIzol reagent (Thermo, 15596018). To analyze the RNA levels of SARS-CoV-2 in infected tissues, quantitative real-time PCR was performed. In brief, 1 μg total RNA was reverse transcribed using ReverTra Ace qPCR RT Kit (TOYOBO, FSQ-101) to produce cDNA with random primers. Reactions of qPCR were carried out using the 2× RealStar Green Power Mixture (Genstar, A311) according to the instruction. The qPCR primers for viral RNA were as follows: THU-2816 (5′-CGATCTCTTGTAGATCTGTTCTC-3′) and THU-2818 (5′-TCAGGGTCAGCTTGCCGTAG-3′). The sequences of the qPCR primers for GAPDH were as follows: GAPDH F (5′-AGGTCGGTGTGAACGGATTTG-3′) and GAPDH R (5′-TGTAGACCATGTAGTTGAGGTCA-3′). All data were normalized relative to the housekeeping gene *GAPDH*.

### Western blotting

Sodium dodecyl sulfate-polyacrylamide gel electrophoresis (SDS-PAGE) immunoblotting was conducted as follows: after trypsinization and cell pelleting at 1500 rpm for 10 min, whole-cell lysates were harvested in cell lysis buffer (50 mM Tris-HCl (pH 7.5), 150 mM NaCl, 1% NP-40, 1 mM EDTA) supplemented with protease inhibitor cocktail (Sigma, P8340). Lysates were electrophoresed in 4%–12% polyacrylamide gels and transferred onto PVDF membrane. The blots were blocked at room temperature for 0.5 h using 5% nonfat milk in 1× phosphate-buffered saline (PBS) containing 0.1% (v/v) Tween 20. The blots were exposed to primary antibodies anti-HA (ABclonal, AE008), anti-ACE2 (Proteintech, 21115-1-AP), and anti-GAPDH (ABclonal, AC001) in 5% nonfat milk in 1× PBS containing 0.1% Tween 20 for 2 h. The blots were then washed in 1× PBS containing 0.1% Tween 20. After 1 h exposure to Alexa Fluor 680-conjugated goat anti-mouse or Alexa Fluor 800-conjugated goat anti-rabbit secondary antibody, and immunoblots were visualized using the ODYSSEY Infrared Imaging System (LI-COR biosciences, USA).

### Mouse challenge experiments

For intranasal infection, animals were housed in an isolator in BSL-2 animal facilities at the Institute of Microbiology, Chinese Academy of Sciences. Mice were intranasally infected with 1 × 10^5^ TCID_50_ of SARS-CoV-2 GFP/ΔN. Mice were then weighed and monitored daily, and sacrificed on day 4 to 7 post infection for serum collection and tissue processing.

### Histopathological analysis

Mouse tissues were excised and fixed with 10% neutral buffered formalin, dehydrated, and embedded in paraffin. Each embedded tissue was sectioned into 5-μm  thickness longitudinal sections. Three tissue sections derived from different parts of each tissue were stained with H&E according to standard procedures for examination by light microscopy. The slides were scanned by Leica Aperio VESA8 equipped with a 40× objective lens (HC Plan-Apochromat; Leica) and acquired a camera (Grasshopper 3 color Bayer Camera, Point Grey) at RT. Images were analyzed byusing Aperio ImageScope software (v12.3.2.7001) and aligned by Adobe Illustrator (CS4).

### Immunohistochemistry

Paraffin-embedded tissues were sectioned at the thickness of 5-μm for immunohistochemistry staining. Sections were deparaffinized and rehydrated, endogenous peroxidases were inactivated with methanol containing 0.3% hydrogen peroxide for 30 min. Antigen retrieval was performed with citrate buffer (pH 6) at 95 °C for 30 min. After incubation in blocking solution (5% normal goat serum) for 10 min at room temperature, the slides were incubated with anti-CD3 antibody (Invitrogen, 1:200), anti-CD20 antibody (Abcam, 1:200) and rabbit anti-CD68 polyclonal antibody (Abcam, 1:200) overnight at 4 °C. After three washes, the sections were incubated with biotinylated anti-IgG at 37 °C for 1 h, followed by Streptavidin-peroxidase conjugate (Zhongshan Biotechnology). Immunoreactivity was detected using 3, 30 diaminobenzidine and the sections were counterstained with hematoxylin for observation by microscopy.

### SARS-CoV-2 GFP/ΔN trVLP preparation

Assembly of a full-length SARS-CoV-2 GFP/ΔN cDNA: the different fragments were obtained by PCR and then digested with restriction enzyme^23^. Digested fragments were purified by E.Z.N.A gel extraction kit (Omega), and were ligated by T4 DNA ligase. Full-length assembly cDNA was extracted using phenol/chloroform, precipitated using isopropanol, and resuspended in 10 μL nuclease-free water.

RNA in vitro transcription, electroporation, and virus production: RNA transcript was in vitro transcribed by the mMESSAGE mMACHINE T7 Transcription Kit (ThermoFisher Scientific) in 30 μL system with some modifications. Twenty micrograms of viral RNA and 20 μg N mRNA were mixed and added to a 4-mm cuvette containing 0.4 mL of Caco-2-N cells (8 × 10^6^) in Opti-MEM. Single electrical pulse was given with a GenePulser apparatus (Bio-Rad) with setting of 270 V at 950 μF. GFP signal can be observed 17 h post electroporation. Three days post electroporation, P0 virus was collected and Caco-2-N cells were infected with P0 virus to amplify virus. The supernatants were collected, passed through a 0.45-μm filter, aliquoted, and frozen at –80 °C refrigerator.

### Intra-testicular inoculation

Mice were housed on a 12 h light/dark cycyle with free water and food provided at a temperature of 22/23 °C, 50% humidity. The procedure were followed by the protocol in a previous study^52^ based on the introduction of fluid through the efferent ductules. All surgical procedures were carried out under aseptic conditions. Ketamine/medetomidine was used for anesthesia induction, and buprenorphine was used for long-acting analgesia after surgery. Following surgery, atipamezole was given to rapidly reverse the sedative effects of medetomidine. The lower abdomen was shaved and swabbed with chlorhexidine. A small incision was made in the lower abdominal and muscle wall slightly to the left of the midline. The testes and epididymis were delivered to a sterile, moist cotton swabs and kept moist with sterile saline during the surgery. Under a dissecting microscope, the efferent ducts were identified and isolated. A hypodermic needle was used to make a small incision in an efferent duct, close to the rete testis, which was then cannulated with a glass microneedle syringe loaded with viral particles. Gentle, steady pressure was applied to ensure the seminiferous tubules were filled and this was monitored by the addition of trypan blue (0.08% (w/v)) to the solution of viral particles. For each testis, injection amount was no more than 15 μL. The testis was carefully replaced into the body cavity, which was closed with both internal and external vicryl (Ethicon) sutures. The mouse was kept warm and observed frequently during the post-operative period. The left testis was treated with SARS-CoV-2 GFP/ΔN and the right testis was treated with PBS as a paired control.

## Supplementary information

Supplementary information
