# Supplementary material for: A tissue specific-infection mouse model of SARS-CoV-2
Source: Cell Discov. 2023 Apr 20;9:43. doi: 10.1038/s41421-023-00536-0 (PMC10117269; doi:10.1038/s41421-023-00536-0)
Supplement: Supplementary file 1 — Supplementary information [file 41421_2023_536_MOESM1_ESM.pdf]

Supplementary information

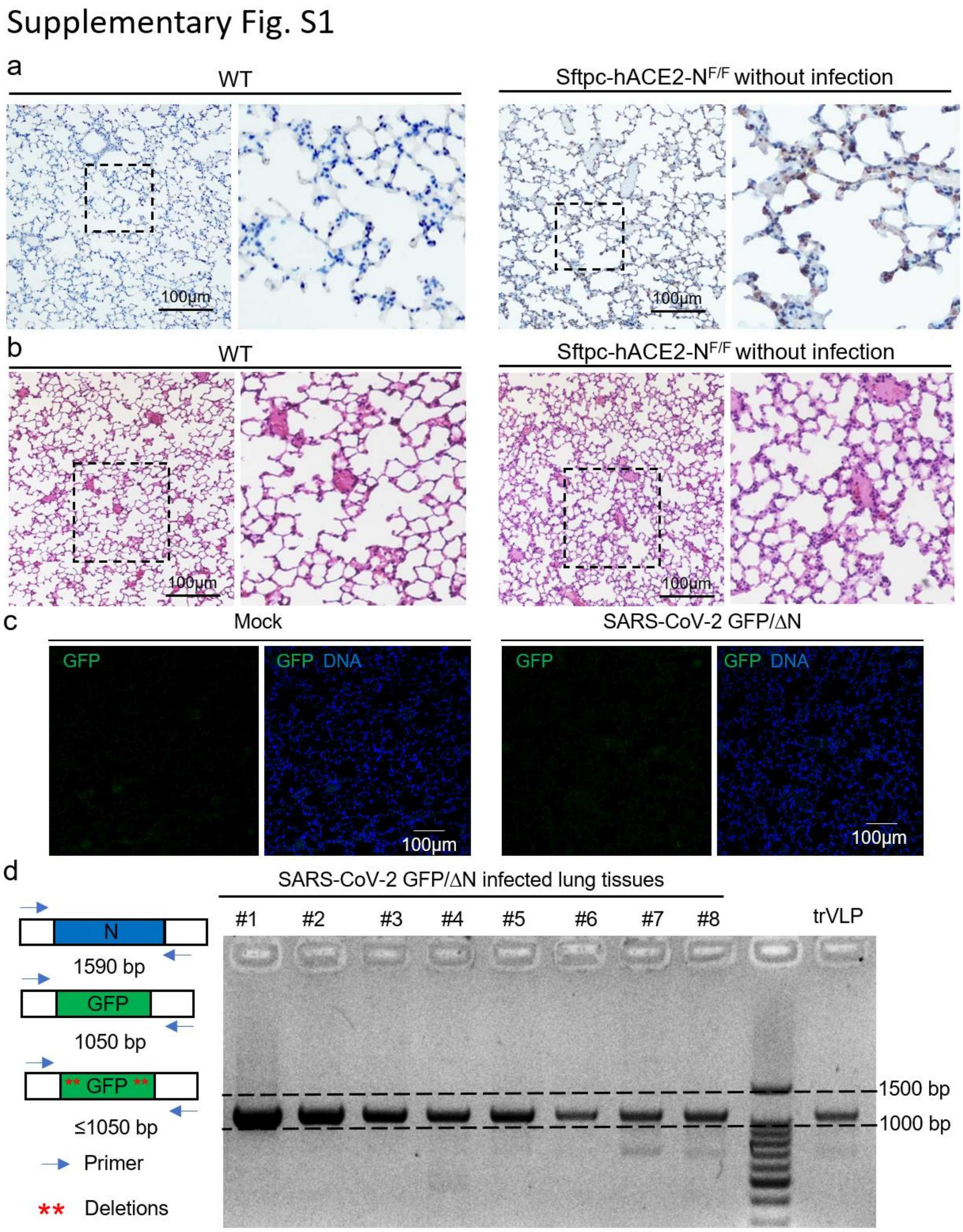

Supplementary Fig. S1 Characterization of the genetic stability of SARS-CoV-2 GFP/ΔN trVLP in mouse

a Immunohistochemistry analysis of SARS-CoV-2 N in mouse lung paraffin sections with staining for anti-HA antibody. b H&E staining shows the pathological changes in Sftpc-hACE2-$N^{F/F}$ mouse lung without SARS-CoV-2 GFP/ΔN infection. c Immunofluorescence analysis of hACE2 mouse lung paraffin sections with staining for anti-GFP antibody (green), which infected by the SARS-

CoV-2 GFP/ΔN trVLP from mouse lung tissue. d RT-PCR was performed with a primer pair flanking the N region of ORF8 and 3’UTR. The PCR products were analyzed by electrophoresis.

Supplementary Fig. S2

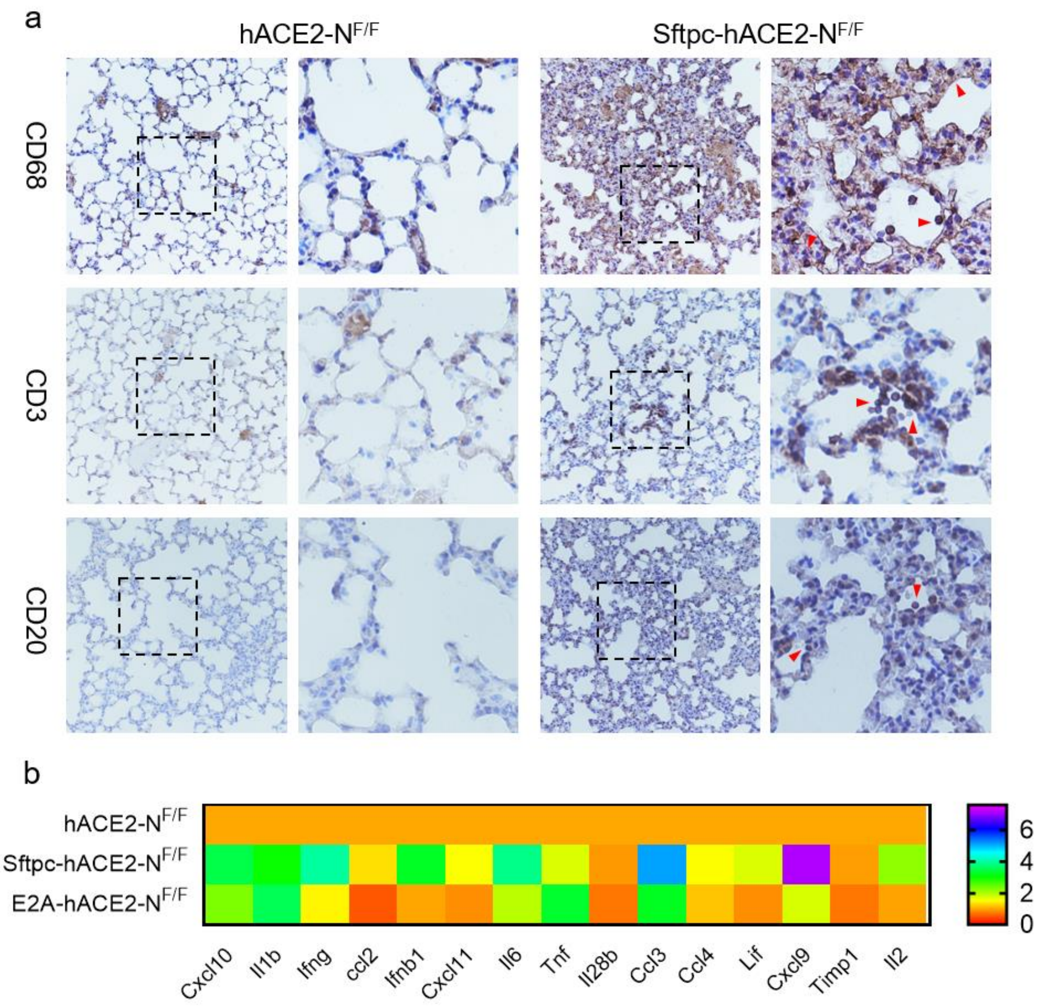

Supplementary Fig. S2 The Sftpc-hACE2-NF/F mice developed interstitial pneumonia upon SARS-CoV-2 GFP/ΔN infection

a IHC staining analysis for $CD68^+$, $CD3^+$ and $CD20^+$ cells in SARS-CoV-2-GFP/ΔN infected mice.

b Cytokine mRNA levels in the SARS-CoV-2- GFP/ΔN infected mice.

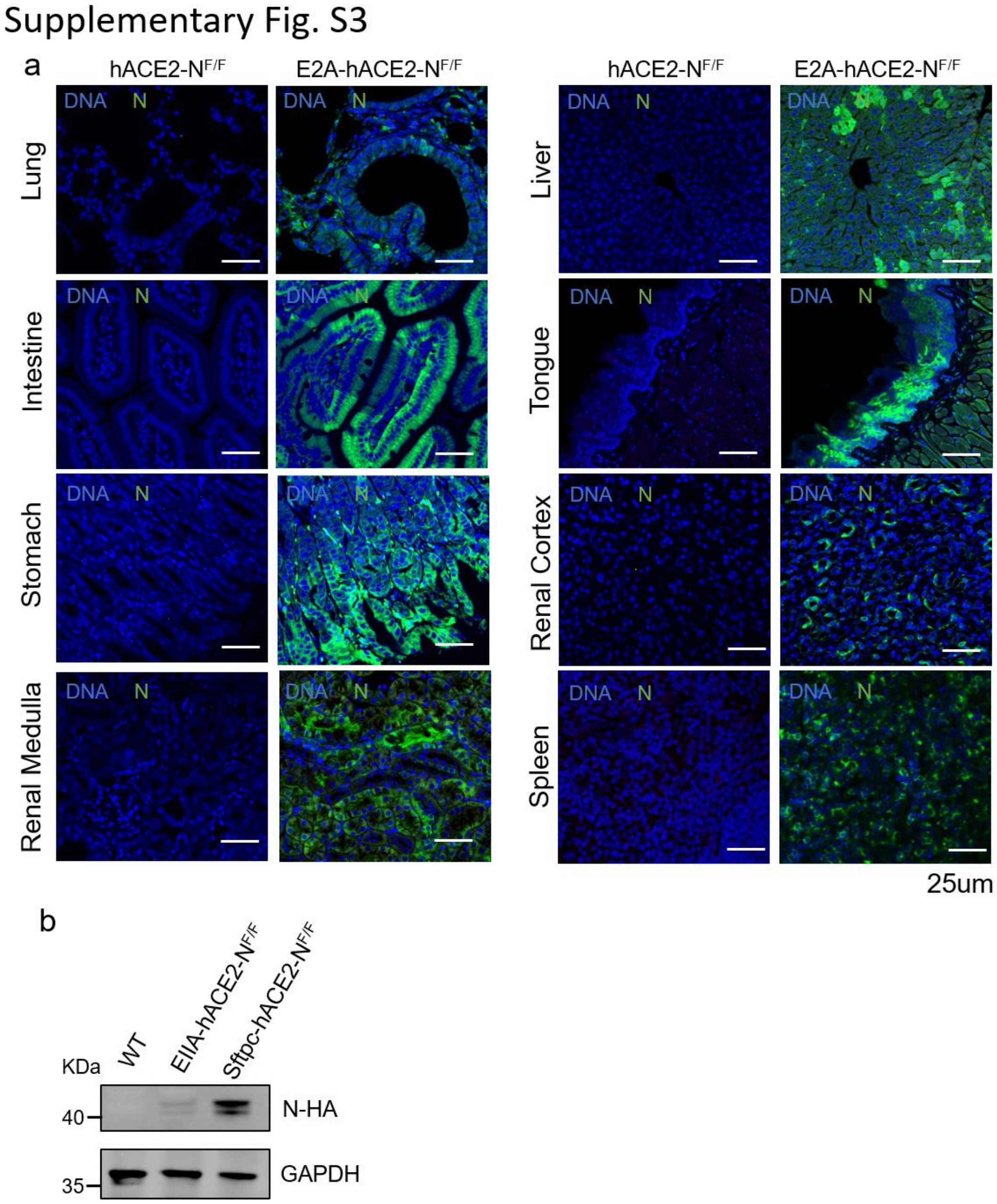

Supplementary Fig. S3 SARS-CoV-2 N protein expressed in a wide range of tissues in EIIa-hACE2-N$^{F/F}$

a Immunofluorescence staining of mouse different tissues paraffin sections for SARS-CoV-2 N protein (green) and DAPI (blue). b Mouse lung tissue lysates were subjected to western blot with anti-HA antibody.

Supplementary Fig. S4

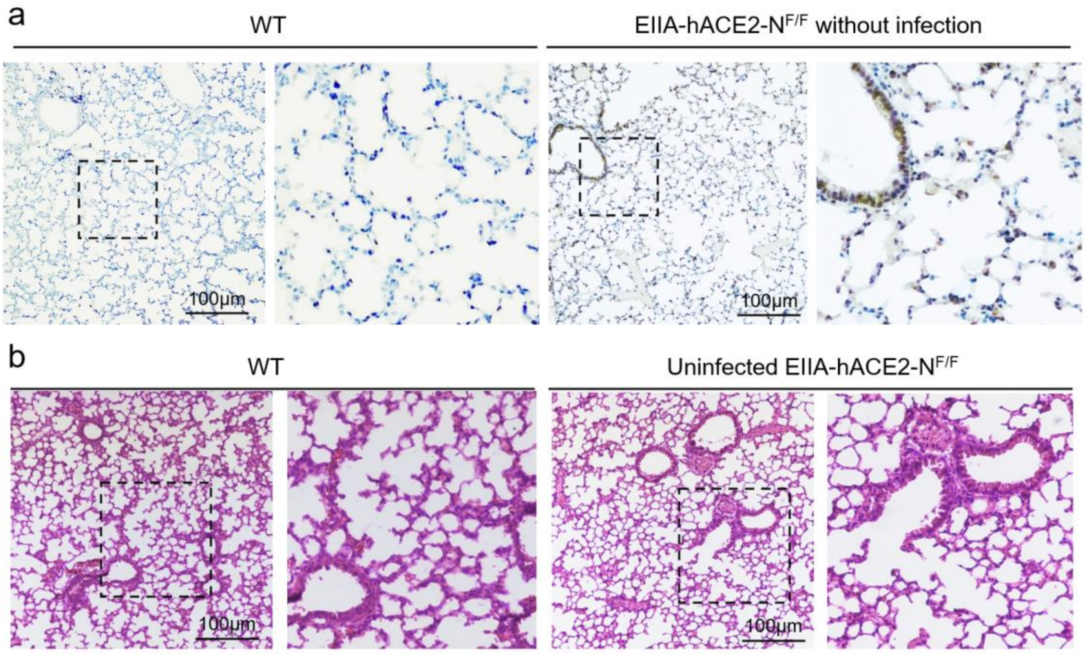

Supplementary Fig. S4 SARS-CoV-2 N protein expressed in lung tissues in EIIa-hACE2-N$^{F/F}$
a Immunohistochemistry analysis of SARS-CoV-2 N in mouse lung paraffin sections with anti-HA antibody. b H&E staining shows the pathological changes in EIIA-hACE2-N$^{F/F}$ mouse lung without SARS-CoV-2 GFP/ΔN infection.

Supplementary Fig. S5

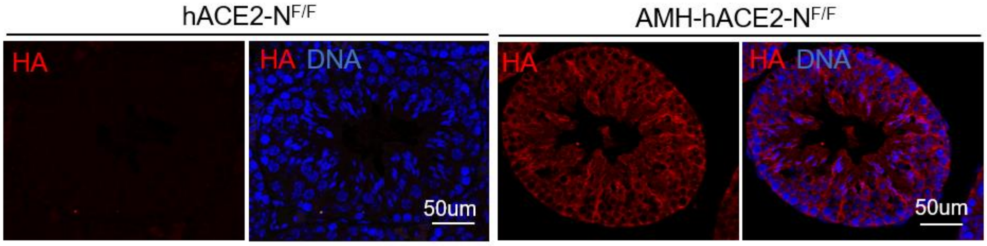

Supplementary Fig. S5 the AMH-hACE2-N$^{F/F}$ mice expresses SARS-CoV-2 N in Sertoli cells
Immunofluorescence staining of mouse testis paraffin sections for SARS-CoV-2 N-HA protein (red) and DAPI (blue).

Supplementary Fig. S6

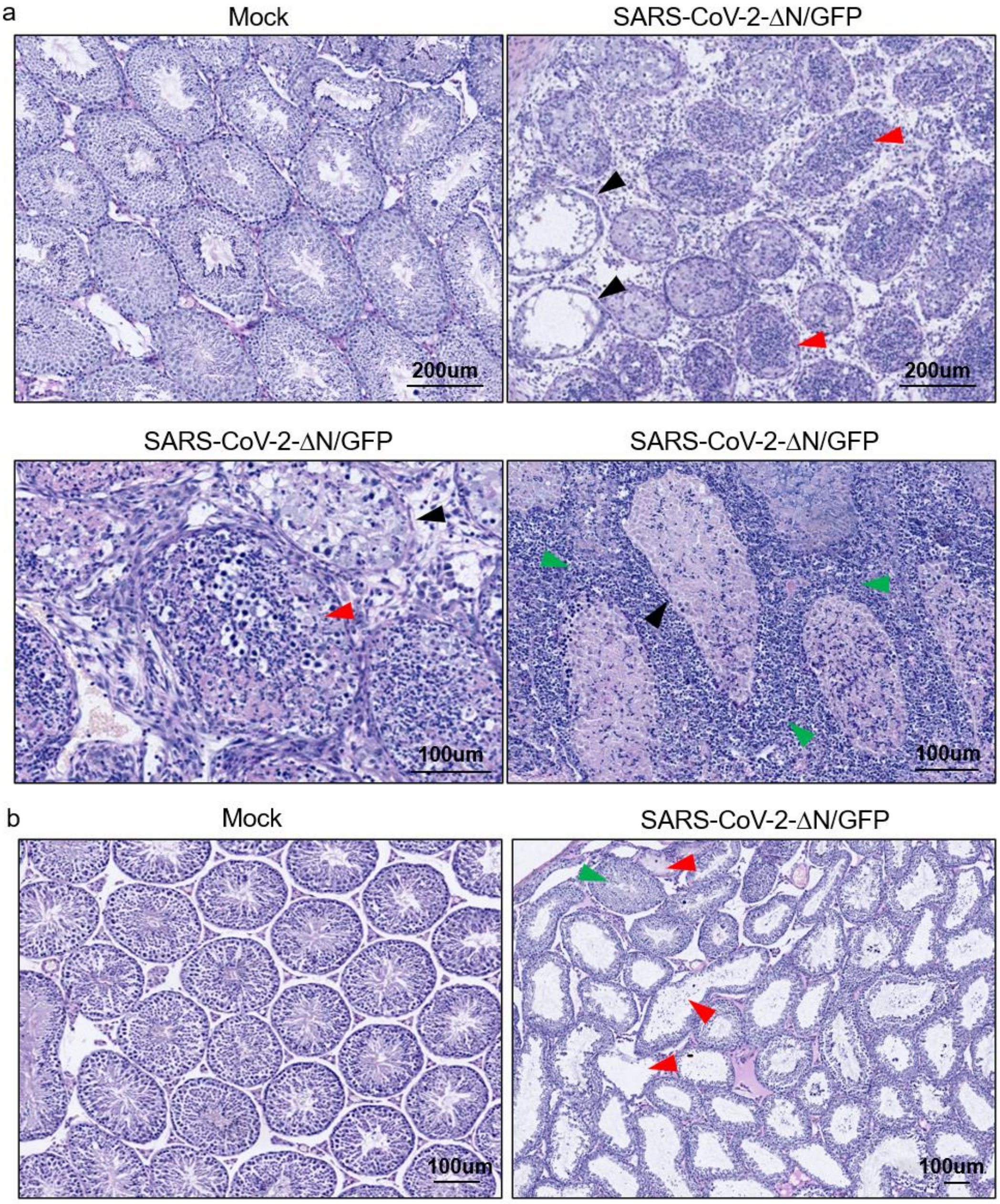

Supplementary Fig. S6 The interstitial mononuclear cell infiltration in SARS-CoV-2 GFP/ΔN-infected testis

a, b Histopathological changes of testis from SARS-CoV-2 GFP/ΔN-infected AMH-hACE2-$N^{F/F}$ mice or control mice. In figure A the dark arrows show the strong damage of the seminiferous tubule structures with loss of the central ductal lumen. The green arrows show the mononuclear cell infiltration. The red arrows show the strong damage of the seminiferous tubules. In figure B the green arrows show the undamaged seminiferous tubules. The red arrows show the damage of the seminiferous tubules.

Supplementary Fig. S7

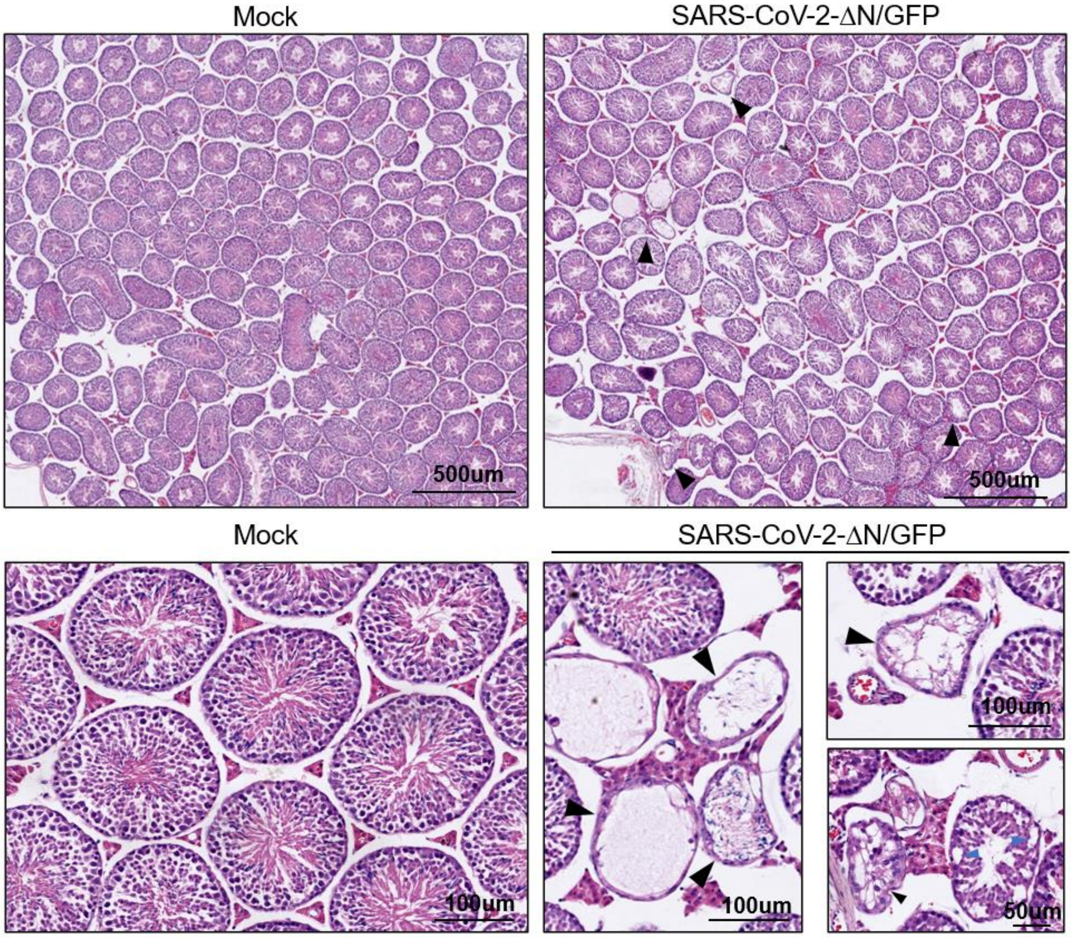

Supplementary Fig. S7 Testicular damage induced by SARS-CoV-2 infection

Histopathological changes of testis from SARS-CoV-2 GFP/ΔN-infected AMH-hACE2-$N^{F/F}$ mice or control mice. Dark arrows show the damage of the seminiferous tubule.

Supplementary Fig. S8

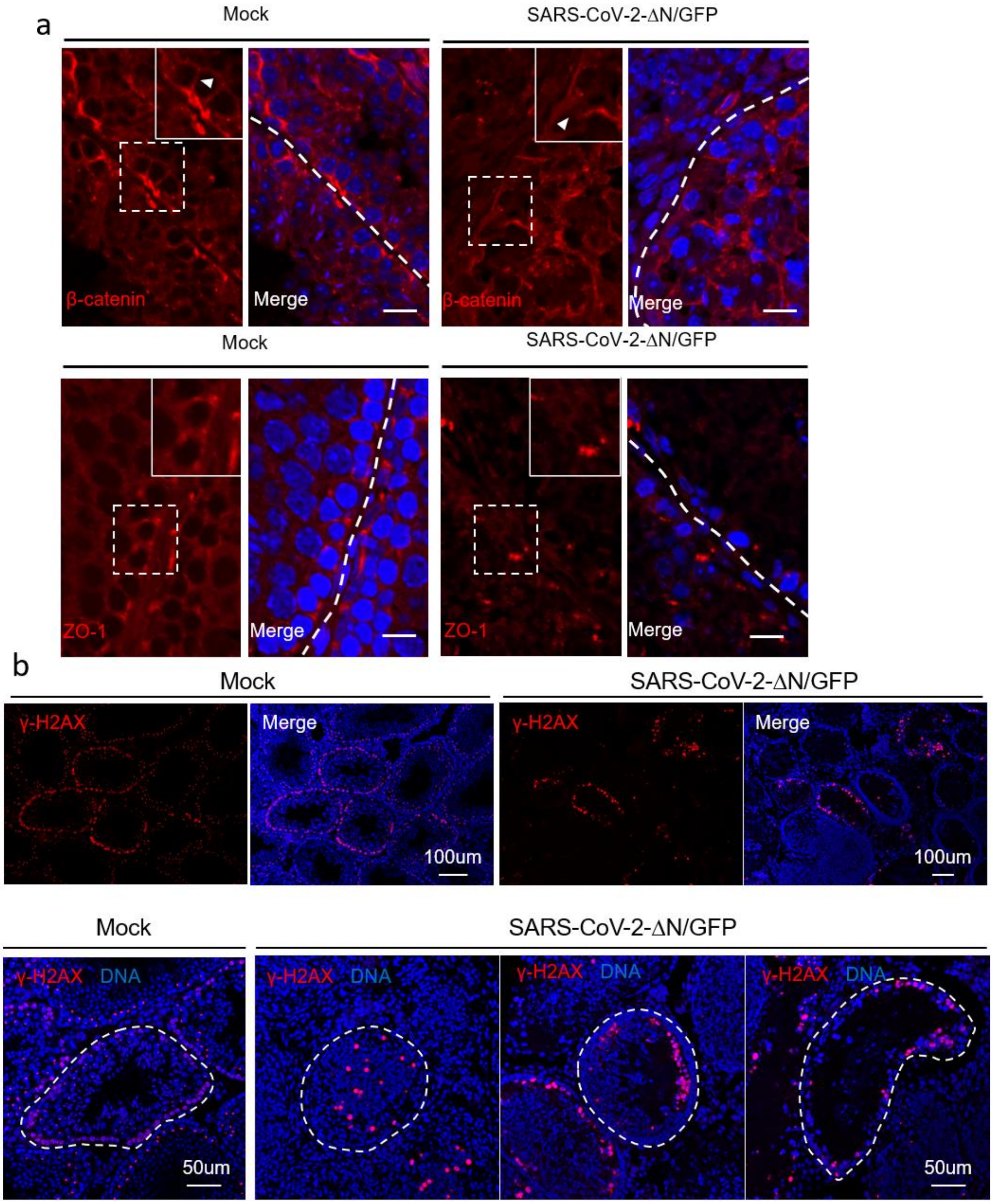

Supplementary Fig. S8 SARS-CoV-2 infection disrupts the integrity of BTB

a Immunofluorescence staining of mouse testis paraffin sections for β-catenin, ZO-1(red) and DAPI (blue). b Immunofluorescence staining of mouse testis paraffin sections for γ-H2AX(red) and DAPI (blue).
